# Tetrodotoxin (TTX): What do we currently know? The state of the art

**DOI:** 10.1007/s00204-026-04548-z

**Published:** 2026-10-12

**Authors:** Łukasz Niżnik, Kamil Kuca, Eugenie Nepovimová, Kamil Jurowski

**Affiliations:** 1 Department of Regulatory and Forensic Toxicology, Institute of Medical Expertises in Łódź, ul. Aleksandrowska 67/93, 91-205 Łódź, Poland; 2 Centre for Basic and Applied Research, Faculty of Informatics and Management, University of Hradec Kralove, CZ-500 03 Hradec Kralove, Czech Republic; 3 Biomedical Research Center, University Hospital Hradec Kralove, Hradec Kralove, Czech Republic; 4 Center of Advanced Innovation Technologies, VSB-Technical University of Ostrava, 708 00 Ostrava-Poruba, Czech Republic; 5 Department of Chemistry, Faculty of Science, University of Hradec Kralove, 50003 Hradec Kralove, Czech Republic; 6 Department of Toxicology, Faculty of Medicine, Collegium Medicum, Rzeszów University, Al. mjr. W. Kopisto 2a, 35-959 Rzeszów, Poland; 7 Laboratory of Toxicological Research and Analyzes, Center for Innovative Research in Medical and Natural Sciences, Faculty of Medicine, Medical College, Rzeszów University, Al. mjr. W. Kopisto 2a, 35-959 Rzeszów, Poland

**Keywords:** Tetrodotoxin, TTX, Neurotoxin, Analytical toxicology, Marine biotoxins, Forensic toxicology

## Abstract

Tetrodotoxin (TTX) is one of the most potent naturally occurring neurotoxins and continues to attract considerable scientific, clinical, forensic, and regulatory attention. Historically associated with pufferfish poisoning in East and Southeast Asia, TTX has recently emerged as a global food safety concern due to its detection in an increasing number of marine and terrestrial organisms and its expanding geographical distribution, including European coastal waters. Despite decades of research, important questions regarding its biosynthesis, ecological origin, toxicokinetics, and environmental dissemination remain unresolved. This review provides a comprehensive overview of current knowledge on TTX. The chemical structure, physicochemical properties, proposed biosynthetic origin, and diversity of naturally occurring TTX analogues are discussed. Attention is devoted to the toxin’s mechanism of action, which involves high-affinity binding to voltage-gated sodium channels and subsequent inhibition of sodium ion conductance, leading to neuromuscular paralysis and potentially fatal respiratory failure. Current evidence regarding toxicokinetics, tissue distribution, elimination pathways, and the suitability of different biological matrices for laboratory confirmation of exposure is also summarized. The review further examines the analytical challenges associated with TTX determination, including its high polarity, occurrence at trace concentrations, rapid elimination from blood, and frequent co-occurrence with structurally related analogues. The evolution of analytical methodologies is presented, from early bioassays and chromatographic techniques with fluorescence or UV detection to advanced liquid chromatography–mass spectrometry platforms, high-resolution mass spectrometry, capillary electrophoresis, immunochemical assays, and emerging biosensor-based approaches. Finally, current regulatory perspectives and risk assessment strategies are discussed in the context of the increasing occurrence of TTX in seafood and the growing need for harmonized monitoring programs. Collectively, this review summarizes the current state of the art, identifies key knowledge gaps, and highlights future directions in TTX research.

## Introduction

Tetrodotoxin (TTX, C_11_H_17_N_3_O_8_; molecular weight 319.27 g/mol) is one of the most potent naturally occurring relatively low-molecular-weight neurotoxins known to date and remains a compound of considerable scientific, clinical, and regulatory interest. Since its initial isolation from pufferfish in the early twentieth century (Katikou et al. 2022), TTX has attracted sustained attention owing to its exceptional toxicity, unique chemical structure, and highly selective mechanism of action involving voltage-gated sodium channels. By blocking sodium influx in excitable tissues, TTX disrupts nerve impulse conduction and skeletal muscle function, potentially leading to respiratory paralysis and death (Zhang 2024). Clinically, TTX intoxication may progress within minutes to hours from perioral paraesthesia and gastrointestinal symptoms to lingual numbness, motor impairment, generalized flaccid paralysis, respiratory failure, cardiac dysrhythmias, unconsciousness and death; importantly, no specific antidote is available. Although a minimum lethal dose of 2 mg in humans has often been cited in the literature, EFSA did not identify primary evidence supporting this value (EFSA Panel on Contaminants in the Food Chain (CONTAM) et al. 2017). Human case reports indicate that serious effects may occur at approximately 0.2 mg, corresponding to ~ 4 µg/kg body weight (bw), and acute poisoning has been associated with estimated exposures in the range of 4–42 µg/kg bw or higher; however, uncertainties in actual exposure reconstruction preclude the use of these human data for acute reference dose derivation. Route-dependent experimental data further illustrate the high acute potency of TTX, with i.p./s.c. LD_50_ values in mice of approximately 9–12.5 µg/kg bw and substantially higher oral/intragastric LD_50_ values of 232–532 µg/kg bw. Based on an acute oral mouse study, EFSA derived a group ARfD of 0.25 µg/kg bw for TTX and its analogues, using 25 µg/kg bw as the reference point and applying a standard uncertainty factor of 100. Together, these data underscore the narrow margin between exposure and life-threatening toxicity, while also highlighting persistent uncertainties regarding human dose–response relationships, analogue potency, toxicokinetics and interspecies extrapolation. Despite decades of research, many aspects of its ecological origin, biosynthesis, toxicokinetics, and global distribution remain incompletely understood (Cembella and Durán-Riveroll 2021).

Historically, tetrodotoxin poisoning has been primarily associated with the consumption of pufferfish in East and Southeast Asia (Noguch and Arakawa 2008). However, increasing evidence indicates that the apparent geographical and taxonomic occurrence of TTX has broadened considerably, partly reflecting improved surveillance and analytical sensitivity during recent decades (Katikou et al. 2022). Furthermore, reports of TTX contamination in European coastal waters and commercially harvested seafood have raised concerns regarding the emergence of new food safety hazards outside regions traditionally affected by TTX intoxication (Antonelli et al. 2022). Environmental changes, alterations in marine ecosystems, species migration, and the complex interactions between toxin-producing microorganisms and host organisms have all been proposed as factors contributing to this expanding distribution (Otero and Silva 2022).

From a clinical and forensic perspective, TTX poisoning continues to represent a significant diagnostic challenge. The absence of a specific antidote, the rapid onset of neurological symptoms, and the relatively short detection window in blood specimens complicate both patient management and laboratory confirmation of exposure (Iwasaki et al. 2014). Accurate identification of TTX in biological specimens is therefore essential for clinical diagnosis, toxicological investigations, and forensic casework. However, analytical determination of TTX remains demanding due to its high polarity, low molecular weight, occurrence at trace concentrations, and the frequent co-existence of structurally similar analogues within complex biological and food matrices (Leung et al. 2011).

Consequently, substantial efforts have been devoted to the development of increasingly sensitive and selective analytical methodologies. While early investigations relied primarily on bioassays and chromatographic methods with fluorescence detection, recent years have witnessed a transition toward advanced liquid chromatography–mass spectrometry platforms, high-resolution mass spectrometry, immunochemical approaches, biosensors, and other emerging technologies. These methodological advances have significantly improved the ability to detect, identify, and quantify TTX and its analogues in clinical, forensic, environmental, and food samples, thereby enhancing both exposure assessment and risk evaluation.

Beyond its toxicological significance, TTX has become an important regulatory challenge. Unlike many established marine biotoxins, TTX emerged relatively recently as a concern for European food safety authorities. This has stimulated the development of monitoring programs, harmonized analytical methodologies, and risk-assessment frameworks aimed at protecting public health. The growing involvement of regulatory agencies, including EFSA (EFSA Panel on Contaminants in the Food Chain (CONTAM) et al. 2017), underscores the increasing importance of TTX not only as a subject of basic toxicological research but also as a toxin of practical relevance for food control, environmental monitoring, and public health preparedness.

The public-health relevance of TTX has changed substantially over the last decade. Historically, human intoxications were associated mainly with pufferfish consumption in Japan and other East and Southeast Asian countries, where fugu and related seafoods are culturally and commercially important. However, TTX is no longer confined to this classical scenario. Global retrospective analyses have documented thousands of seafood-associated TTX poisoning cases across five continents (EFSA Panel on Contaminants in the Food Chain (CONTAM) et al. 2017), and recent reports from Europe, the Mediterranean region, the Middle East and North Africa indicate that locally sourced seafood can also become a source of exposure. In parallel, TTX and its analogues have been detected in European bivalves and gastropods, including mussels, oysters, clams and trumpet shells, shifting the toxin from a geographically familiar hazard to an emerging issue for seafood surveillance and risk assessment.

Several converging factors make TTX particularly relevant now: the apparent expansion of TTX-positive organisms beyond traditional endemic regions, the occurrence of TTX in commercially relevant European shellfish, evidence of seasonal and environmental drivers such as seawater temperature, and unresolved questions regarding the biological origin, trophic transfer and accumulation of the toxin. These developments are occurring in a regulatory context in which TTX is still not managed with the same maturity as many established marine biotoxins, despite the EFSA-derived guidance value of 44 µg TTX equivalents/kg shellfish meat and repeated calls for harmonised monitoring, certified reference materials and validated analytical methods.

Hence, the present review critically synthesizes current evidence on TTX as an emerging toxicological, analytical and regulatory challenge. Particular emphasis is placed on its chemical characteristics and biological origin, mechanisms of toxicity, toxicokinetic uncertainties, clinically and forensically relevant matrices, analytical strategies for qualitative and quantitative determination, and implications for seafood surveillance and public-health risk assessment. By integrating experimental, clinical, forensic, analytical and environmental evidence, this review aims to identify where current knowledge is sufficient for risk management and where key uncertainties still prevent robust, evidence-based control of TTX-related risks.

## Methods

### Search for publications on Tetrodotoxin

The search strategy was developed through systematic refinement, integrating controlled vocabulary with free-text terminology to maximize both sensitivity and specificity. The primary search centred on major classical bibliographic databases, including PubMed, Scopus, and Google Scholar. To enhance coverage and identify materials not consistently indexed across these platforms, supplementary screening was performed using AI-assisted literature searching tools. Their use served to detect potentially overlooked studies, identify grey literature or rapidly evolving evidence, and confirm the completeness of conventional database retrieval. This was particularly valuable given the multidisciplinary nature of tetrodotoxin research, spanning chemistry, toxicology, clinical medicine, environmental sciences and analytical methodology.

Search terms were constructed around the central toxin (“tetrodotoxin”, “tetrodotoxins”, “TTX”, “TTX analogs”), complemented by terminology reflecting structural and biochemical context, toxicological outcomes, and analytical or diagnostic approaches (e.g., “LC-MS”, “LC-MS/MS”, “liquid chromatography”, “HPLC”, “high-resolution mass spectrometry”, “capillary electrophoresis”, “GC-MS”, “GC-MS/MS”, “immunoassay”, “biosensor”). No restrictions regarding language or publication date were applied. Studies providing primary data on TTX or any structurally defined analog were considered eligible if they addressed at least one of the following domains: chemical or biochemical characterisation; mechanism of action or toxicodynamics; clinical toxicology, including case reports and case series; animal or cellular models; analytical determination and method validation; diagnostic applications; forensic investigations; or regulatory and risk-assessment frameworks. All primary research designs were included, encompassing experimental, observational and analytical-method development studies. Studies were excluded when TTX was mentioned only incidentally, when full text could not be retrieved, or when methodological detail was insufficient for extraction.

All records retrieved from bibliographic databases were exported to reference-management software, where duplicates were removed before screening. Titles and abstracts were screened against predefined eligibility criteria, followed by full-text assessment of potentially relevant articles. Disagreements or uncertain cases were resolved by discussion among the authors.

### Chemistry and origin of TTX

As was mentioned in introduction, TTX (C_11_H_17_N_3_O_8_; molecular weight 319.27 g/mol) is relatively small molecule but is highly oxygenated guanidinium alkaloid distinguished by an unusual molecular architecture characterized by a rigid dioxaadamantane cage structure, heavily substituted with heteroatoms and exhibiting substantial stereochemical complexity. The molecule incorporates an ortho acid functionality and a cyclic guanidinium hemiaminal group, and contains nine consecutive stereogenic centers, among which is a quaternary bridgehead carbon bearing a nitrogen atom (Chen et al. 2024). The guanidinium group confers strong basicity with pKa value of 8.76 (Durán-Riveroll and Cembella 2017), while the extensive hydroxylation results in pronounced polarity and high aqueous solubility (Qian et al. 2024), with negligible solubility in non-polar organic solvents (Alcaraz et al. 1999).

From a biogenetic and ecological perspective, TTX is remarkable for its broad phylogenetic distribution, having been detected in numerous and often unrelated marine and terrestrial taxa including pufferfish (Wassel et al. 2024; Zhang et al. 2025a), rough-skinned (Gall et al. 2022) and fire-bellied newts (Kudo et al. 2015), blue-ringed octopus (Whitelaw et al. 2019; Zhang et al. 2023), xanthid crabs (Zhang et al. 2023, 2025b), gastropods (keshavarz lelekami et al. 2023; Han et al. 2023), flatworms (Yonezawa et al. 2024), and frogs (Tanu et al. 2001). In most marine systems, current evidence favours exogenous acquisition through bacterial production or trophic transfer rather than de novo synthesis by the host animal, however, the complete biosynthetic pathway and the relative contribution of host-associated microbiota remain unresolved (Lago et al. 2015; Zhang et al. 2024). In aquatic organisms, TTX-producing or TTX-associated bacteria have been reported from several genera, including *Vibrio* (Bacchiocchi et al. 2021), *Pseudoalteromonas* (Chau et al. 2021), *Shewanella* (Auawithoothij and Noomhorm 2012), *Aeromonas* (Liu et al. 2015) and *Raoultella* (Yu et al. 2011), among others (Magarlamov et al. 2017). In parallel, feeding experiments showed loss of toxicity under TTX-free diets and reacquisition after exposure to TTX-containing prey, which strongly support an exogenous acquisition model, followed by bioaccumulation and tissue-specific sequestration (Noguchi et al. 2006). Nevertheless, bacterial involvement should be interpreted with caution, because reproducible de novo TTX production in culture and the genetic basis of TTX biosynthesis have not been conclusively resolved.

Among TTX-bearing taxa, high toxin concentrations are frequently reported in reproductive tissues, particularly ovaries and eggs (Melnikova and Magarlamov 2022), where it is thought to act as a chemical defence, reducing predation risk during vulnerable early developmental stages. In some pufferfish species, TTX has also been proposed to function as a a pheromone-like chemoattractant or reproductive cue involved in reproductive communication and spawning behavior (Matsumura 1995; Williams 2010).

In contrast, the origin of TTX in terrestrial amphibians has been more contentious: some studies, such as that by Lehman et al. (Lehman et al. 2004), failed to detect TTX-producing symbionts at relevant body sites, suggesting endogenous synthesis, whereas Vaelii et al. (Vaelli et al. 2020), demonstrated, through combined microbiome analyses and culture-based work in rough-skinned newts (*Taricha granulosa*), that several skin-associated bacterial strains (*Aeromonas*, *Pseudomonas*,* Shewanella*, and *Sphingopyxis*) are capable of producing authentic TTX under in vitro conditions. The study conducted by Yotsu-Yamashita et al. (Yotsu-Yamashita et al. 2012) demonstrated that, although belonging to the same species of red-spotted newt (*Notophthalmus viridescens*), individuals from different geographic regions exhibit variation in TTX presence and concentration. Moreover, the authors showed that specimens maintained on a -free diet progressively lose their toxicity, further supporting the influence of environmental factors on toxin accumulation. Study of Horie et al. (Horie et al. 2026) lead to a complementary discovery of deoxy and epi-deoxy TTX analogues in *Cynops ensicauda popei*, which supports a stepwise oxidative pathway on a preformed TTX/10-hemiketal-type scaffold, suggesting that bacterial symbiosis or dietary acquisition may interact with lineage-specific late-stage biotransformation to shape TTX analogue profiles. These findings suggest partial conservation of late-stage oxidative steps, but also differences in overall biosynthetic diversification between terrestrial and marine taxa. Thus, the amphibian data are particularly important because they show that the “bacterial origin versus endogenous synthesis” dichotomy is probably too simplistic. TTX occurrence may instead reflect a continuum of microbial production, environmental acquisition, host sequestration and taxon-specific chemical modification.

Several naturally occurring and semi-synthetic tetrodotoxin (TTX) analogues have been described in the literature, reflecting epimerization, oxidation, deoxygenation, dehydration, lactonization, and other structural modifications of the parent toxin, especially: 4-epi-TTX (Yasumoto and Yotsu-Yamashita 1996), 6-epi-TTX (Yotsu-Yamashita et al. 2012), 9-epi-TTX (Yaegashi et al. 2022), dehydration products such as 4,9-anhydro-TTX (Hassoun et al. 2022), 4,4a-anhydroTTX (Saruhashi et al. 2016), oxidized, e.g. 11-oxoTTX (Yotsu-Yamashita et al. 2012) and deoxygenated, e.g. 5,11-dideoxyTTX (Yotsu-Yamashita et al. 2013), 11-deoxyTTX (Yang et al. 1992) analogs were also identified in specific taxa. However, for some reported analogues, unambiguous curated chemical identifiers, such as SMILES, InChI, CAS numbers, or database-deposited structures, are not currently available in commonly used chemical databases. Therefore, Table 1. summarizes only those TTX analogues for which a chemical structure or reliable chemical identifier could be verified and represented with sufficient confidence. This approach was adopted to avoid speculative reconstruction of highly stereochemically complex TTX congeners.

**Table 1:** Structurally verified tetrodotoxin (TTX) analogues and congeners with available chemical identifiers

Name	Structure	Chemical identifiers
4-EpiTTX		SMILES: C([C@@]1([C@H]2[C@@H]3[C@@H](N = C(N[C@@]34[C@@H]([C@@H]1O[C@]([C@H]4O)(O2)O)O)N)O)O)O CAS: 98242-82-1
6-EpiTTX		SMILES: C([C@]1([C@H]2C3[C@H](N = C(N[C@]34[C@@H]([C@](O2)(O[C@H]1C4O)O)O)N)O)O)O CAS: 112318-40-8
5-deoxyTTX		SMILES: C1C2C([NH+] = C(NC23C(C(C1(CO)O)[C@H](C(= O)O3)O)O)N)O
11-deoxyTTX		SMILES: C[C@@]1([C@H]2[C@@H]3[C@@H](N = C(N[C@]34[C@@H]([C@](O2)(O[C@H]1C4O)O)O)N)O)O CAS: 113564-23-1
5,11-dideoxyTTX		SMILES: O[C@@]1(C)C[C@@H]2[C@@H](O)NC(= N)N[C@@]32[C@@H](O)C(= O)O[C@H]1[C@@H]3O
6,11-dideoxyTTX		SMILES: C[C@@H]1[C@@H]2O[C@]3(O)O[C@H]1 C(O)[C@@]1(NC(= N)N[C@H](O)[C@@H]21)[C@@H]3O
8,11-dideoxyTTX		SMILES: C[C@@]1(O)[C@@H]2 C[C@]34NC(= N)N[C@H](O)[C@H]3[C@H]1O[C@](O)(O2)[C@H]4O
1-hydroxy-5,11-dideoxyTTX		SMILES: ON1C(= N)N[C@H](O)[C@H]2 C[C@@](O)(C)[C@H]3 °C(= O)[C@H](O)[C@]21[C@H]3O
11-oxoTTX		SMILES: C12C3C(C4C(C1(C(C(O3)(O4)O)O)NC(= NC2O)N)O)(C(O)O)O CAS: 123665-88-3
5,6,11-trideoxyTTX		SMILES: C[C@@H]1 C[C@@H]2[C@H](N = C(N[C@@]23[C@@H]([C@@H]1 °C(= O)[C@H]3O)O)N)O
8-epi-5,6,11-trideoxyTTX		SMILES: O=C1O[C@H]2[C@H](O)[C@@]3(NC(= N)N[C@H](O)[C@H]3 C[C@H]2 C)[C@@H]1O
11-norTTX-6,6-diol		SMILES: O[C@]12O[C@H]3[C@@H](O)[C@@]4(NC(= N)N[C@H](O)[C@H]4[C@@H](O1)C3(O)O)[C@@H]2O
11-norTTX-6(R)-ol		SMILES: [C@H]12[C@@H]3[C@@H]([C@@H]4[C@H]([C@]1([C@H]([C@](O3)(O4)O)O)NC(= N[C@@H]2O)N)O)O CAS: 81520-41-4
11-norTTX-6(S)-ol		SMILES: [C@H]12[C@@H]3[C@@H]([C@@H]4 C([C@]1([C@@H]([C@](O3)(O4)O)O)NC(= N[C@@H]2O)N)O)O CAS: 156336-07-1
Chiriquitoxin		SMILES: C12C3C(C4C(C1(C(C(O3)(O4)O)O)NC(= NC2O)N)O)(C(C(C(= O)O)N)O)O CAS: 61132-15-8
4,9-anhydroTTX		SMILES: C([C@]1([C@@H]2[C@H]([C@@]34[C@H]5[C@@](O2)(OC1C3C(O5)N = C(N4)N)O)O)O)O CAS: 13072-89-4
Tetrodonic acid		SMILES: C([C@@]1([C@@H]([C@@H]2[C@H]3 N = C(N[C@]2([C@H]([C@H]1O)O)[C@@H](O3)C(= O)O)N)O)O)O CAS: 3270-35-7

It should be noted that the list presented in Table 1 does not exhaust all TTX-related analogues reported in the literature. Several additional congeners have been described in isolation, biosynthetic, analytical, or structural studies, but their curated chemical structures or machine-readable identifiers are not readily available in major chemical databases. These include, among others, 9-epiTTX, 9-epiTTX-10,8-lactone, 4-epi-5,11-dideoxyTTX, 1-hydroxy-8-epi-5,6,11-trideoxyTTX, 4-epi-5,6,11-trideoxyTTX, TTX-8-O-hemisuccinate, TTX-11-carboxylic acid, 6-epi-4,9-anhydroTTX, 4,9-anhydro-11-deoxyTTX, 4,9-anhydro-5,11-dideoxyTTX, 4,9-anhydro-5,6,11-trideoxyTTX, 4,9-anhydro-8-epi-5,6,11-trideoxyTTX, 4,4a-anhydro-5,6,11-trideoxyTTX, 1-hydroxy-4,4a-anhydro-8-epi-5,6,11-trideoxyTTX, and 4-cysTTX. These compounds have been reported in specific contexts, including total synthesis, biosynthetic pathway studies, isolation from pufferfish, newts, polyclads, and comparative toxicity studies. However, due to the lack of unambiguous curated structural identifiers, they were not included in the structural part of the table. This limitation should be considered when interpreting the structural diversity of TTX analogues.

### Toxicity and mechanism of action

TTX is a highly potent neurotoxin characterized by pronounced acute toxicity in experimental animal models, with clear route-dependent differences in lethality. In the EFSA risk assessment, acute i.p. and s.c. LD_50_ values in mice were reported in the range of approximately 9–12.5 µg/kg bw, whereas substantially higher LD_50_ values were observed after oral/intragastric administration, including 232 µg/kg bw in the study by Abal et al. (Abal et al. 2017) and 532 µg/kg bw in an earlier study (Xu et al. 2003). Abal et al. also identified an acute oral NOAEL of 75 µg/kg bw, although EFSA selected the lower tested dose of 25 µg/kg bw as the reference point for deriving the group ARfD of 0.25 µg/kg bw because of the steep dose–response relationship and uncertainties related to the observation period. More recent controlled mouse studies by Wang et al. (Wang et al. 2023) further support this route-dependent toxicity pattern, reporting an LD₅₀ of 8.6 µg/kg bw after intramuscular administration and 378.7 µg/kg bw after oral gavage, together with dose-dependent and largely reversible loss of muscle strength at sublethal exposures.

Clinically, tetrodotoxin poisoning is characterized by an initial phase of perioral and lingual paresthesia, dizziness, and gastrointestinal symptoms, followed by progressive neuromuscular dysfunction including generalized paresthesia, ataxia, dysarthria, dysphagia, and limb weakness. Severe intoxication may progress to cranial nerve involvement, cardiovascular instability, ascending paralysis, respiratory failure, coma, and death in the absence of timely supportive treatment (Noguchi and Ebesu 2001). Analysis of outbreak investigations and case series suggests that the minimum lethal dose for an adult is on the order of 2–3 mg of TTX. However, EFSA noted that the original source and underlying data for the frequently cited human minimum lethal dose of approximately 2 mg could not be retrieved; therefore, this value should be interpreted with caution.

At the molecular level, TTX binds with high affinity to receptor site 1 in the outer vestibule of sensitive voltage-gated sodium channels (French et al. 2010), where its guanidinium group interacts with residues lining the selectivity filter (Tikhonov and Zhorov 2018), forming a hydrogen-bond network that occludes sodium ion permeation from the extracellular site (Chen and Chung 2014). Rather than penetrating deeply through the pore, the bulky cage-like structure of tetrodotoxin remains at the extracellular mouth of the channel, where it acts as a highly complementary molecular plug that physically occludes the ion-conducting pathway (Choudhary et al. 2003; Fozzard and Lipkind 2010). As a result, Na⁺ ions are unable to access the selectivity filter and permeate through the channel. This pore blockade occurs without directly interfering with channel gating, ultimately preventing action potential initiation and propagation. This mechanism explains the rapid neuromuscular paralysis and respiratory failure observed in severe TTX poisoning.

**Fig. 1:**
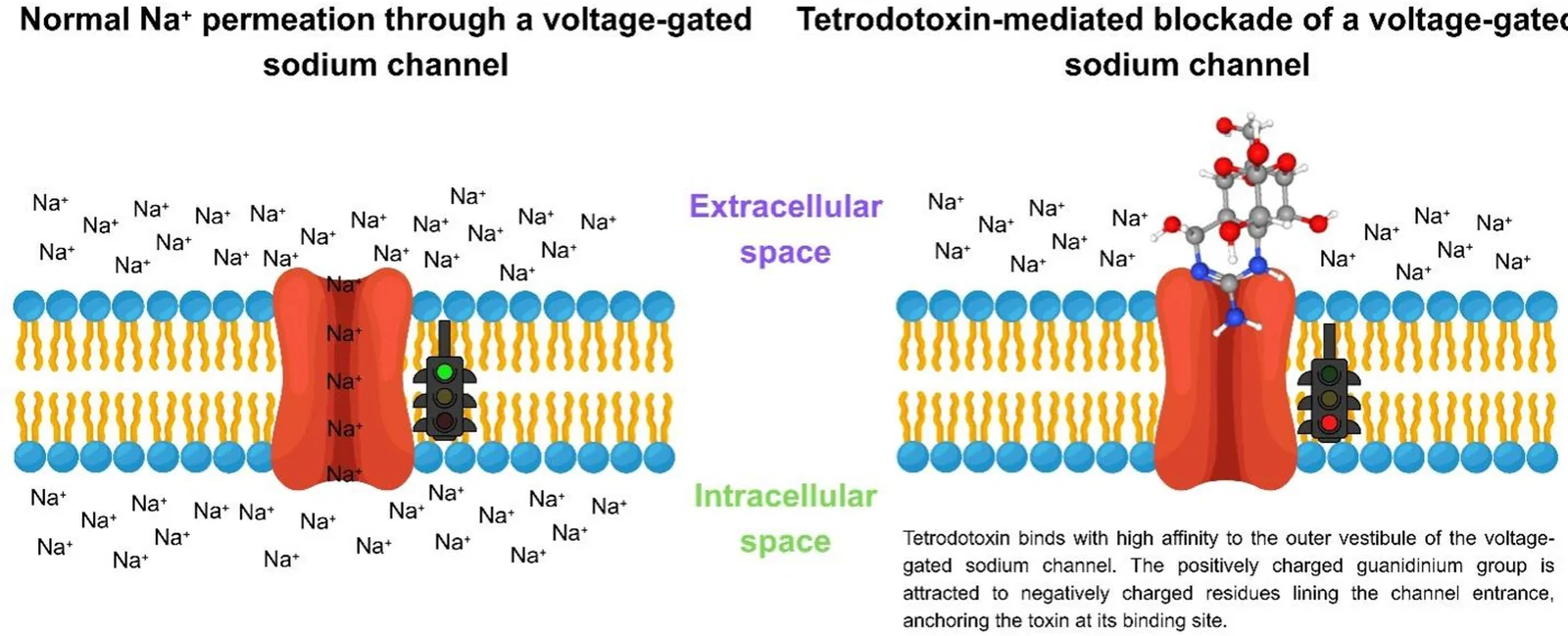
Visual representation of the molecular mechanism of action of TTX

This interaction yields half-maximal inhibition in the low-nanomolar range for so-called TTX-sensitive voltage-gated sodium channels (Nav) isoforms (Nav1.1, Nav1.2, Nav1.3, Nav1.4, Nav1.6 and Nav1.7) (Li et al. 2023), whereas channels such as the predominant cardiac isoform Nav1.5 and peripheral Nav1.8/1.9 are relatively TTX-resistant (Shen et al. 2018). Systematic characterization of TTX analogues by Tsukamoto et al. (Tsukamoto et al. 2017), has revealed that subtle modifications can markedly alter binding affinity to individual Nav subtypes, indicating that naturally occurring congener mixtures may exhibit distinct toxic potency profiles and raising the possibility that differential Nav isoform selectivity contributes to the spectrum of clinical presentations observed in different species and exposure contexts. EFSA similarly distinguished nanomolar sensitivity of Nav1.1, Nav1.2, Nav1.3, Nav1.4, Nav1.6 and Nav1.7 from the much lower sensitivity of Nav1.5, Nav1.8 and Nav1.9. Visualisation of TTX toxicity is represented in form of figure (Fig. 1).

### Samples of biological specimens

Clinical and experimental evidence indicates that TTX is rapidly absorbed following exposure and distributes efficiently into the systemic circulation and highly perfused tissues. In a study using radiolabeled 11-[³H]TTX conducted by Hong et al. (Hong et al. 2017) in Sprague–Dawley rats, peak plasma radioactivity was observed within approximately 10 min after intramuscular administration, followed by rapid elimination with a plasma half-life of about 2.3 h. Radioactivity was no longer detectable in plasma after 24 h, whereas elevated levels persisted in the stomach wall, intestine, lungs, and kidneys, indicating extensive tissue distribution and preferential accumulation within gastrointestinal and renal compartments. Subsequent physiologically based kinetic (PBK) modelling studies by Noorlander et al. (Noorlander et al. 2022) in rats and mice indicated that hepatic metabolism contributes only minimally to TTX clearance. Consistent with experimental observations showing that less than 10% of the toxin is metabolized, PBK simulations demonstrated that systemic elimination is governed predominantly by renal excretion, with active tubular transport representing a major component of total renal clearance. Clinical pharmacokinetic data provided by Kavoosi et al. (Kavoosi et al. 2020) indicate that TTX is characterized by predictable and dose-proportional systemic exposure. In healthy adults receiving subcutaneous doses of 15–45 µg TTX, peak plasma concentrations were achieved within approximately 1.5 h, and both C_max_ and Area under curve increased proportionally with dose. Elimination was relatively rapid, with a terminal half-life ranging from 4.3 to 4.6 h and plasma concentrations falling below the limit of quantification in most participants within 24 h after administration (Hong et al. 2017). From a diagnostic perspective, both blood/plasma and urine may be useful biological specimens, but their interpretation depends strongly on the timing of collection. TTX appears to remain detectable in plasma for a relatively short period, usually less than 24 h, whereas urine may remain positive for several days after ingestion and is therefore often considered the preferred matrix for confirmation of poisoning when food remnants are unavailable. Observations from human poisoning cases further highlight the diagnostic value of urinary TTX measurements. While the toxin is generally detectable in blood, serum, or plasma for only 12–36 h following ingestion, it may remain measurable in urine for at least four days and, in some cases, up to five days after exposure (Bane et al. 2014). Consequently, urine is considered the most sensitive and practical biological matrix for laboratory confirmation of TTX poisoning (O’Leary et al. 2004; Leung et al. 2011). This observation is supported by a fatal pufferfish poisoning case described by Noguchi et al., (Noguchi et al. 2011) in which urinary TTX concentrations (2 MU/mL) exceeded those measured in blood (0.7 MU/mL). Taken together, these observations highlight the importance of specimen selection in TTX analysis and demonstrate that the diagnostic window varies considerably among biological matrices, with urine providing the longest period for reliable toxin detection. However, urinary concentrations should be interpreted cautiously because they are influenced by hydration status, urine output, and the timing of sample collection; creatinine adjustment is therefore recommended when quantitative interpretation is attempted.

### Analytical challenges in TTX detection in clinical and forensic samples

Analytical detection of TTX in clinical, forensic, environmental, and food samples remains particularly challenging because of a combination of physicochemical properties of the toxin and limitations associated with sample collection and instrumental analysis. Although many of these analytical challenges are shared across all sample types, the present section focuses primarily on issues relevant to clinical and forensic biological specimens. One of the primary obstacles is the highly polar nature of TTX and many of its naturally occurring analogues or congeners. Because of its extreme polarity, TTX exhibits poor retention on conventional reversed-phase columns, often leading to inadequate separation and compromised analytical performance (Leung et al. 2011). To overcome these limitations, methods commonly employ hydrophilic interaction liquid chromatography (HILIC) or ion-pairing strategies (Fong et al. 2011). Although these approaches generally improve retention and sensitivity, each presents distinct practical limitations. HILIC methods require careful control of column re-equilibration to ensure stable retention times and reproducible chromatographic performance (McCalley 2018), whereas ion-pairing reagents may reduce compatibility with mass spectrometric detection by causing ion suppression, contaminating the analytical system, and prolonging column equilibration and system stabilization (Annesley 2003). Consequently, the development of reliable analytical procedures for TTX continues to require careful optimization of both chromatographic and detection parameters. In addition, the toxin possesses a relatively low molecular mass and is often present at extremely low concentrations in highly heterogeneous food and clinical matrices (Hu et al. 2022). Consequently, analytical methods must provide exceptional sensitivity to reliably detect and quantify trace amounts of TTX in complex matrices. Although bioassays and immunochemical methods remain valuable tools for rapid screening, their performance may be affected by matrix effects, limitations in analyte and matrix applicability, and the risk of false-positive or false-negative results. As a result, positive findings generally require confirmation by chromatographic methods coupled to mass spectrometry, with LC–MS/MS remaining the most widely accepted confirmatory approach despite its greater analytical complexity and more demanding sample preparation requirements (Tsagkaris et al. 2019). Further complications arise from the toxicokinetic characteristics of TTX. Following exposure, circulating concentrations may decline rapidly, resulting in analyte levels that approach or fall below the detection limits of less sensitive techniques (Hong et al. 2017). Consequently, a negative blood or plasma result, particularly when sampling is delayed, should not be considered sufficient to exclude TTX poisoning. Whenever available, urine, gastric contents, vomitus, and suspected food remnants should be prioritized, as these matrices may remain informative even when circulating TTX concentrations are no longer detectable. Moreover, biological and food samples contain numerous endogenous and exogenous compounds that can interfere with extraction efficiency, chromatographic separation, and mass spectrometric detection (Peng et al. 2016; Golubović et al. 2019). Therefore, rigorous optimization of sample preparation procedures and comprehensive method validation are essential. Validation should include the use of matrix-matched calibration, appropriate internal standards, assessment of recovery, matrix effects, carry-over, analyte stability, limits of detection and quantification, precision, accuracy, chromatographic retention time, and ion transition or ion ratio criteria to ensure reliable analytical results, particularly in forensic toxicology.

The determination of TTX is additionally complicated by the frequent co-occurrence of structurally related analogues and congeners in natural samples (Kudo et al. 2012). Many of these compounds differ only slightly in their molecular structure and often exhibit similar fragmentation patterns and closely related mass-to-charge ratios. In addition, the limited availability of certified reference standards for many TTX analogues complicates their unequivocal identification and accurate quantification. Furthermore, incomplete knowledge regarding the relative toxic potencies of individual analogues hampers toxicological interpretation (Rambla-Alegre et al. 2018; Reverté et al. 2024), particularly when multiple congeners are detected simultaneously. Consequently, their chromatographic separation, unequivocal identification, and accurate quantification remain challenging, particularly when multiple analogues are present at trace concentrations within complex biological or food matrices.

Beyond the analytical challenges encountered in clinical specimens, forensic toxicology presents additional difficulties related to the diversity and condition of post mortem materials. Depending on the circumstances of death and the available material, TTX analysis may be performed using peripheral or cardiac blood (Cho et al. 2012), urine (Fong et al. 2011), gastric contents (Namba et al. 2022), tissue samples (Waters et al. 2018), and residual food suspected to be the source of intoxication. Delays between death and sample collection, post mortem degradation, autolytic changes, and the potential influence of post mortem redistribution may further complicate analyte detection and interpretation (Galera and Rossato-Grando 2026). Therefore, careful specimen selection, appropriate storage and handling, validated analytical procedures, and strict maintenance of the chain of custody are essential to ensure the reliability and evidential value of forensic toxicological findings. These analytical challenges have driven the continuous development of increasingly sensitive and selective methodologies for the detection, identification, and quantification of TTX and its analogues.

### Analytical approaches for tetrodotoxin detection and quantification

Early clinical determination of TTX relied predominantly on high-performance liquid chromatography (HPLC) with fluorescence detection (FLD), initially developed for environmental samples and later adapted to human matrices. Onoue et al. (Onoue et al. 1983) first demonstrated HPLC–fluorescence separation of TTX and paralytic shellfish poisons in fish and shellfish, defining optimal excitation and emission conditions (332 nm for excitation, and 453 nm for emission) for routine fluorometric detection. Building on this concept, Chen and Chou (Chen and Chou 1998) applied post-column alkaline degradation to generate a fluorescent derivative in shellfish and puffer fish, providing a robust environmental toxicology assay that became the basis for subsequent clinical adaptations. In human urine, Kawatsu et al. (Kawatsu et al. 1999) were the first to combine immunoaffinity chromatography with post-column fluorescence HPLC, achieving a low limit of detection (2 ng/ml) and showing that TTX remains detectable in urine up to seven days after fugu ingestion. O’Leary et al. (O’Leary et al. 2004) later introduced a clinical HPLC–fluorescence method with post-column alkaline derivatization, detecting TTX in urine from all seven poisoned patients and in one serum sample. Authors achieved minimum quantifiable TTX concentrations of 5 ng/mL for serum samples and 20 ng/mL for urine samples. Compared with fluorescence-based methods, pure UV/DAD detection has historically been uncommon for native TTX because of its weak chromophore, but both post-column and pre-column derivatization strategies have been used to enable chromatographic detection by UV/DAD. Yu et al. (Yu et al. 2010) proposed a rapid HPLC–UV assay with C18/WCX solid-phase extraction and creatinine correction, enabling routine screening of urine and plasma within approximately 24 h after exposure. In this study the detection limit for TTX standard, urine and plasma were 10 ng/ml. A recent study by Kawasue et al. (Kawasue et al. 2024) introduced a genuinely UV-based reversed-phase HPLC method using pre-column derivatization of the vic-diol moiety of TTX with a newly designed boron reagent (NBD-H-PBA), detectable by conventional UV. This RP-HPLC–UV (Reversed–phase high–performance liquid chromatography with ultraviolet detection) workflow, intended for rapid assessment of outbreak samples including food remnants, achieved a limit of detection (LOD) of 0.06 mg/kg in fish flesh, demonstrating that with appropriate derivatization, TTX can be quantified by standard RP-HPLC–UV without MS. Liquid chromatography–mass spectrometry (LC–MS) and tandem mass spectrometry (LC–MS/MS) have become the principal analytical platforms for TTX determination in both clinical and food matrices, offering substantially improved sensitivity, selectivity, and structural confirmation compared with earlier chromatographic approaches. The first LC–MS methods for TTX were reported in the early 2000s by Shoji et al. (Shoji et al. 2001) By using liquid chromatography electrospray ionization mass spectrometry (LC ESI-MS), authors systematically characterized the fragmentation patterns of eight tetrodotoxin analogs including 4-epiTTX, 4,9-anhydroTTX, 6-epiTTX, 11-deoxyTTX, 5-deoxyTTX, and 11-norTTX derivatives. In this study authors also utilized LC-FLD method in which they achieved a LOD of 0.4 pmol. In another study Horie and co-workers (Horie et al. 2002b), applied LC ESI-MS to pufferfish tissues. Using a Shodex RSpak NN-414 column with an ammonium acetate–methanol mobile phase and positive-ion electrospray detection, TTX was monitored as the protonated molecular ion (m/z 320) in selected-ion monitoring (SIM) mode. The method exhibited linearity over the range of 0.01–1.0 µg/mL, recoveries of 77.7–80.7% from fish tissue extracts, and a LOD of approximately 0.1 µg/g. A parallel study of Horie et al. (Horie et al. 2002a) employing a TSK-gel ODS (Octadecylsilane) 80Ts reversed-phase column with heptafluorobutyric acid as an ion-pairing reagent achieved a calibration range of 0.1–1.0 µg/mL and a LOD of 1 µg/g in fish tissue. One of the earliest LC–MS/MS applications for biological samples was reported by Tsai et al. (Tsai et al. 2006), who described a method involving ultrafiltration and C18 cartridge cleanup followed by LC–MS. The method exhibited a LOD of 15.6 nM, with TTX concentrations ranging from 4.5 to 40.6 nM in blood and from 47 to 344 nM in urine. In another study Jen et al. (Jen et al. 2008), analyzed serum from human poisoning cases using solid-phase extraction (SPE) coupled with triple-quadrupole mass spectrometry operating in positive-ion multiple-reaction monitoring (MRM) mode. The method demonstrated linearity between 1 and 100 ng/mL, with LOD and limit of quantification (LOQ) of approximately 0.1 and 1.0 ng/mL, respectively. TTX concentrations of 3.30 ± 0.08 ng/mL were measured in patient serum samples. Similarly, Fong and co-workers (Fong et al. 2011) developed a high-throughput double-SPE HPLC–MS/MS method for plasma and urine, combining C18 and HILIC cleanup with ion-pair chromatography with creatinine normalization for urinary TTX quantification. The assay achieved a LOD of 0.13 ng/mL and an LOQ of 2.5 ng/mL in both matrices, with linearity extending to 500 ng/mL in urine and 20 ng/mL in plasma. Application to eight poisoning cases revealed urinary TTX concentrations ranging from 17.6 to 460.5 ng/mL, whereas plasma samples remained below the LOD. It is also worth noting that creatinine-normalized urinary levels (7.4–41.1 ng µmol⁻¹) correlated with poisoning severity. Using hydrophilic interaction liquid chromatography coupled with tandem mass spectrometry (HILIC-MS/MS), Nzoughet et al. (Nzoughet et al. 2013) established a validated method for TTX analysis in gastropods. The assay employed matrix-matched calibration to compensate for matrix effects and achieved LOD and LOQ values of 7.3 and 24.5 µg/kg, respectively. Recovery ranged from 103% to 112%, and analyte confirmation was based on two MRM transitions (m/z 319.92→302.00 and 319.92→161.80), demonstrating the applicability of HILIC-MS/MS for sensitive and reliable determination of TTX in seafood matrices. To facilitate rapid clinical diagnosis of TTX intoxication, Saito et al. (Saito et al. 2014) introduced a simplified LC–MS/MS method employing MonoSpin CBA and MonoSpin amide extraction cartridges for serum and urine, respectively. The method eliminated solvent evaporation steps and reduced sample preparation time to approximately 10–15 min. Using HILIC separation and tandem mass spectrometric detection, the assay achieved LODs of 0.5 ng/mL in serum and 0.25 ng/mL in urine, with recoveries of 86.5–93.4% and matrix effects below 10%. Using a validated UPLC-MS/MS method, Vlamis et al. (Vlamis et al. 2015) investigated the occurrence of TTX in shellfish harvested from Greek coastal waters. Analysis of 84 samples collected between 2012 and 2014 identified TTX in several mussel samples at concentrations up to 194 µg/kg. The study demonstrated the applicability of UPLC-MS/MS for sensitive detection of TTX in complex shellfish matrices and provided the first evidence of TTX contamination in Greek shellfish, raising concerns regarding the emergence of this toxin in European marine environments. In another study Tsujimura and Yamanouchi (Tsujimura and Yamanouchi 2015) developed a rapid and sensitive LC–MS/MS method for the determination of tetrodotoxin in human serum using only 0.1 mL of sample. The method employed ultrafiltration sample preparation, a multi-mode Scherzo SS-C18 column, and MRM with the transition m/z 320→162 for TTX and voglibose as an internal standard. The assay demonstrated excellent linearity over the concentration range of 0.5–200 ng/mL (*r* = 0.9994), recoveries of 91.0–110.8%, and a LOQ of 0.5 ng/mL. Application of the method to two confirmed cases of pufferfish poisoning revealed maximum serum TTX concentrations of 30.2 and 56.1 ng/mL, respectively, allowing toxin monitoring throughout the clinical course and demonstrating the utility of LC–MS/MS for both diagnosis and toxicokinetic investigations of human TTX intoxication Bane et al. (Bane et al. 2016) developed an LC–HRMS method for the identification and quantification of tetrodotoxin and several of its analogues in pufferfish and molluscan shellfish. When evaluated in TTX-free mackerel extracts fortified with tetrodotoxin, the method achieved an LOD of 0.041 µg/g and an LOQ of 0.136 µg/g, demonstrating its suitability for the detection of naturally occurring TTXs and their analogues in seafood matrices. Patria et al. (Patria et al. 2020) developed an UHPLC–HILIC–MS/MS method for the simultaneous determination of paralytic shellfish toxins TTX, and its analogs in bivalves, achieving excellent linearity (R² > 0.99), satisfactory repeatability, and detection limits well below current regulatory action levels. Han et al. (Han et al. 2019) developed a UHPLC–HILIC–MS/MS method using an XBridge BEH (Bridged ethylene hybrid) Amide column combined with mixed-mode cation-exchange SPE cleanup and validamycin as an internal standard. The assay exhibited a linear range of 1.5–1500 µg/kg, recoveries of 82–94%, an LOD of 0.5 µg/kg, and an LOQ of 1.0 µg/kg in gastropod and mollusk tissues. Validamycin effectively compensated for matrix effects and analytical variability. An even greater improvement in sensitivity was reported by Li et al. (Li et al. 2022), who introduced a proton-enhanced electrospray ionization strategy involving post-column formic acid (FA) infusion. Using a UHPLC–MS/MS analysis, this approach achieved exceptionally low detection and quantification limits of approximately 20 and 67 pg/g, respectively, in fish tissue extracts. Calibration remained linear from 1 to 2000 ng/mL, and extraction recoveries approached 90%, enabling reliable quantification of trace TTX concentrations from as little as 0.2 g of tissue. To enable non-destructive TTX monitoring, Meng et al. (Meng et al. 2022) combined in vivo solid-phase microextraction with UPLC-MS/MS analysis. The GO-PAN@PNE fiber coating allowed direct extraction of TTX from living pufferfish muscle, eliminating conventional tissue homogenization and solvent extraction procedures. TTX was analyzed using HILIC-UPLC-MS/MS with MRM transitions of m/z 320→302 and 320→162. The method exhibited good linearity over the range of 150–1000 ng g⁻¹ (R² = 0.9895), with a LOD of 32 ng g⁻¹ and a LOQ of 150 ng g⁻¹ in spiked fish samples. Ye et al. (Ye et al. 2022) developed a sensitive HPLC-MS/MS method for the simultaneous determination of TTX in fresh pufferfish tissues and heat-processed aquatic products. Sample preparation involved extraction with 1% acetic acid in methanol, immunoaffinity clean-up, and HILIC separation on a BEH Amide column coupled to triple-quadrupole MS operated in positive ESI-SRM mode. TTX was monitored using the precursor ion m/z 320.2 and product ions m/z 302.2, 162.2, and 284.2, with the transition 320.2→302.2 selected for quantification. The method exhibited excellent linearity (R² = 0.9998) over the range of 0.2–100 ng g⁻¹, with an LOD of 0.2 ng g⁻¹ and an LOQ of 0.5 ng g⁻¹. Recoveries ranged from 90.5% to 107.2%, demonstrating satisfactory accuracy and precision across multiple aquatic matrices. Using mixed-mode hydrophilic interaction/ion-exchange SPE coupled with LC-MS/MS, Xin et al. (Xin et al. 2022) developed a rapid method for TTX determination in human plasma. The assay required only 100 µL of plasma, eliminated evaporation and ultrafiltration steps, achieved extraction recoveries of approximately 81%, and provided a lower limit of quantification (LLOQ) of 0.1 ng/mL over a linear range of 0.1–20 ng/mL. Zheng et al. (Zheng et al. 2023) developed a UHPLC-Q-Exactive Orbitrap HR-MS method combined with modified QuEChERS extraction for the simultaneous determination of TTX, 4,9-anhydroTTX, and paralytic shellfish toxins in human serum. Separation was achieved on a TSK-Gel Amide-80 HILIC column, while detection was performed in full scan/dd-MS² mode. The method provided LODs and LOQs of 0.67–2.61 ng/mL and 2.23–8.69 ng/mL, respectively, with recoveries ranging from 85.3% to 118.2%, and was successfully applied to clinical samples from poisoned patients. In a recent clinical study, Fang et al. (Fang et al. 2024) developed an LC–MS/MS method using online SPE with a mixed-mode MCX cartridge, achieving a LOD of 0.1 ng/mL and a LOQ of 0.3 ng/mL in both plasma and urine. Sample preparation included automated SPE cleanup, which minimized matrix effects and supported rapid, high-throughput analysis. More recently, a LC–MS/MS method by Fang et al. (Fang et al. 2025) employing first-dimension reversed-phase separation followed by heart-cut transfer to a BEH Amide HILIC column was introduced for poisoned plasma and urine samples. Using kasugamycin as an internal standard, the method achieved linearity between 0.2 and 40.0 µg/L, LOD and LOQ values of 0.3 and 1.0 µg/L, respectively, matrix effects of approximately 81–99%, and intra- and inter-day RSD(Relative standard deviation) below 7.5%.

Table 2 provides an overview of liquid chromatography-based methods used for TTX determination, together with the investigated matrices, sample preparation procedures, internal standards (IS), and key analytical performance parameters, including LOD and LOQ.

**Table 2:** Liquid chromatography-based methods used for TTX determination

Authors	Technique	Matrix	Sample preparation / Column / IS	LOD / LOQ	Comments
Onoue et al. (1983)	HPLC-FLD	Fish and shellfish poisons	N/A	Not reported	Optimal fluorescence: excitation 332 nm; emission 453 nm
Chen and Chou (1998)	HPLC-FLD	Shellfish, pufferfish	Post-column alkaline degradation	Not reported	N/A
Kawatsu et al. (1999)	HPLC-FLD	Human urine	Immunoaffinity chromatography; post-column fluorescence	LOD 2 ng/mL	TTX detectable in urine up to 7 days after fugu ingestion
O’Leary et al. (2004)	HPLC-FLD	Human serum, urine	Post-column alkaline derivatization	MQL 5 ng/mL (serum); 20 ng/mL (urine)	TTX detected in urine from all 7 patients and in 1 serum sample
Yu et al. (2010)	HPLC-UV	Human urine, plasma	C18/WCX SPE; creatinine correction	LOD 10 ng/mL	N/A
Kawasue et al. (2024)	RP-HPLC-UV	Fish flesh	Pre-column vic-diol derivatization with NBD-H-PBA	LOD 0.06 mg/kg	N/A
Shoji et al. (2001)	LC-ESI-MS / LC-FLD	Pufferfish ovary and newt skin	N/A	LOD 0.4 pmol	Characterized fragmentation of TTX and 7 analogues
Horie et al. (2002b)	LC-ESI-MS (SIM)	Pufferfish tissues	Shodex RSpak NN-414; ammonium acetate-methanol	LOD approximately 0.1 µg/g	Linearity 0.01–1.0 µg/mL; recoveries 77.7-80.7%; monitored m/z 320
Horie et al. (2002a)	LC-ESI-MS	Pufferfish tissues	TSK-gel ODS 80Ts; heptafluorobutyric acid ion pairing	LOD 1 µg/g	N/A
Tsai et al. (2006)	LC-MS/MS	Human blood, urine	Ultrafiltration; C18 cartridge cleanup	LOD 15.6 nM	TTX: 4.5-40.6 nM in blood and 47-344 nM in urine
Jen et al. (2008)	LC-MS/MS (MRM)	Human serum	SPE	LOD 0.1 ng/mL; LOQ 1.0 ng/mL	Linearity 1-100 ng/mL; patient serum 3.30 ± 0.08 ng/mL
Fong et al. (2011)	HPLC-MS/MS	Human plasma, urine	Double SPE (C18 and HILIC); ion-pair LC; creatinine normalization	LOD 0.13 ng/mL; LOQ 2.5 ng/mL	Creatinine-normalized levels correlated with severity
Nzoughet et al. (2013)	HILIC-MS/MS	Gastropods	Matrix-matched calibration	LOD 7.3 µg/kg; LOQ 24.5 µg/kg	Recoveries 103-112%; confirmation by two MRM transitions
Saito et al. (2014)	HILIC-MS/MS	Human serum, urine	MonoSpin CBA (serum); MonoSpin amide (urine); no evaporation	LOD 0.5 ng/mL (serum); 0.25 ng/mL (urine)	Preparation 10–15 min; recoveries 86.5-93.4%; matrix effects <10%
Vlamis et al. (2015)	UPLC-MS/MS	Shellfish	Not specified	Not reported	TTX up to 194 µg/kg; first evidence in Greek shellfish
Tsujimura and Yamanouchi (2015)	LC-MS/MS (MRM)	Human serum	Ultrafiltration; Scherzo SS-C18; voglibose IS	LOQ 0.5 ng/mL	Linearity 0.5-200 ng/mL; recoveries 91.0-110.8%; 0.1 mL sample
Bane et al. (2016)	LC-HRMS	Pufferfish, molluscan shellfish	Evaluation in fortified TTX-free mackerel extracts	LOD 0.041 µg/g; LOQ 0.136 µg/g	N/A
Han et al. (2019)	UHPLC-HILIC-MS/MS	Gastropod, mollusk tissues	Mixed-mode cation-exchange SPE; XBridge BEH Amide; validamycin IS	LOD 0.5 µg/kg; LOQ 1.0 µg/kg	Range 1.5–1500 µg/kg; recoveries 82-94%
Patria et al. (2020)	UHPLC-HILIC-MS/MS	Bivalves	N/A	Not specified	N/A
Li et al. (2022)	UHPLC-MS/MS	Fish tissues	Post-column formic acid infusion; proton-enhanced ESI	LOD approximately 20 pg/g; LOQ approximately 67 pg/g	Linearity 1-2000 ng/mL; recoveries approximately 90%; 0.2 g tissue
Meng et al. (2022)	HILIC-UPLC-MS/MS	Living pufferfish muscle	In vivo SPME with GO-PAN@PNE fiber	LOD 32 ng/g; LOQ 150 ng/g	Non-destructive assay; linearity 150-1000 ng/g (R² = 0.9895)
Ye et al. (2022)	HPLC-MS/MS	Fresh pufferfish, heat-processed aquatic products	1% acetic acid in methanol; immunoaffinity cleanup; BEH Amide	LOD 0.2 ng/g; LOQ 0.5 ng/g	Linearity 0.2-100 ng/g (R² = 0.9998); recoveries 90.5-107.2%
Xin et al. (2022)	LC-MS/MS	Human plasma	Mixed-mode HILIC/ion-exchange SPE; no evaporation or ultrafiltration	LLOQ 0.1 ng/mL	Linearity 0.1-20 ng/mL; recoveries approximately 81%; 100 µL plasma
Zheng et al. (2023)	UHPLC-Q-Exactive Orbitrap HRMS	Human serum	Modified QuEChERS; TSK-Gel Amide-80 HILIC	LOD 0.67-2.61 ng/mL; LOQ 2.23-8.69 ng/mL	Full scan/dd-MS²; recoveries 85.3-118.2%; TTX, 4,9-anhydroTTX, and PSTs
Fang et al. (2024)	LC-MS/MS	Human plasma, urine	Automated online SPE; mixed-mode MCX cartridge	LOD 0.1 ng/mL; LOQ 0.3 ng/mL	N/A
Fang et al. (Fang et al. 2025)	2D-LC-MS/MS	Human plasma, urine	RP separation; Amide HILIC; kasugamycin IS	LOD 0.3 µg/L; LOQ 1.0 µg/L	Linearity 0.2–40.0 µg/L; matrix effects 81-99%; RSD <7.5%

N/A – Not applicable

While LC–MS-based techniques currently represent the reference methods for TTX analysis, the toxin’s highly polar and charged structure also makes it suitable to electrophoretic separation. As a result, capillary electrophoresis (CE) and related techniques have been explored as alternative approaches for TTX determination, particularly for rapid separations and toxin profiling. The earliest electrophoretic TTX analysis approach was reported by Shimada et al. (Shimada et al. 1983) in 1983, who employed Shimadzu IR-2 A isotachophoretic analyzer to perform capillary isotachophoresis for the quantification of TTX in puffer fish tissues. In this method, TTX was separated as a cationic analyte using a leading electrolyte consisting of 5 mmol/L potassium acetate (pH 6.0) supplemented with Triton X-100 and dioxane, while the terminating electrolyte contained 10 mmol/L β-alanine adjusted to pH 4.5 with acetic acid. The method enabled direct analysis of crude puffer fish extracts without extensive sample clean-up, and LOD of approximately 0.25 µg TTX was reported. Subsequent studies explored the application of conventional capillary zone electrophoresis (CZE) for TTX determination in fish tissues. Ru and Luo (Ru and Luo 2000) developed an internal-standard-based CZE method for TTX determination in frilled fish samples. The method employed ephedrine hydrochloride as an internal standard to compensate for migration-time variability and improve injection reproducibility. Separation was performed in a fused-silica capillary using an acetate buffer system composed of acetic acid and sodium acetate adjusted to pH 4.0, while detection was carried out by UV absorbance at 200 nm under conventional CZE operating voltages. The incorporation of the internal standard reportedly improved both analytical precision and calibration linearity compared with external calibration alone, with the LOD for TTX reported at 0.001 mg/mL. The most advanced CE-based methodology for TTX analysis was introduced by Beach et al. (Beach et al. 2018) in 2018 through the development of a CE–MS/MS method for the simultaneous determination of multiple polar marine toxins, including tetrodotoxins, paralytic shellfish poisoning toxins, and domoic acid. Separation was achieved using a highly acidic background electrolyte composed of 5 mol/L formic acid, which promoted efficient protonation and on-capillary stacking of the analytes. The CE system was interfaced with a triple-quadrupole mass spectrometer operating in positive-ion multiple-reaction-monitoring mode. The method was validated in mussel tissue extracts and incorporated multiple structurally related internal standards together with matrix-matched calibration to compensate for ionization suppression effects. The reported LOD reached 0.0052 mg/kg for tetrodotoxins in mussel tissue.

The capillary electrophoresis-based methods reported for TTX determination are summarized in Table 3. The table highlights electrophoretic separation approaches, detection systems, analytical performance, and their applicability to marine and biological matrices.

**Table 3:** Capillary electrophoresis-based methods used for TTX determination

Authors	Technique	Matrix	IS / Detection	LOD
Shimada et al. (1983)	Capillary isotachophoresis	Pufferfish tissues	No IS used; Isotachophoretic analyzer (Shimadzu IR-2 A)	0.25 µg
Ru and Luo (2000)	CZE-UV	Frilled fish tissues	Ephedrine hydrochloride IS; UV 200 nm	0.001 mg/mL
Beach et al. (2018)	CE-MS/MS	Mussel tissues	Multiple IS; matrix-matched calibration	0.0052 mg/kg

Gas chromatographic methods for TTX analysis represent an early but now largely historical approach to this toxin. One of the earliest chromatographic approaches for TTX determination was reported by Suenaga and Kotoku (Suenaga and Kotoku 1980), who employed gas chromatography (GC) following alkaline conversion of TTX to the C9-base derivative and trimethylsilylation. The assay achieved a LOD of 0.4 µg per sample and provided recoveries of 83.7% and 65.3% from stomach contents and serum, respectively. To facilitate the detection of TTX in clinical specimens, Kurono et al. (Kurono et al. 2001) established a GC-MS (gas chromatography–mass spectrometry) assay based on SPE, alkaline conversion of TTX to the characteristic C9-base, and trimethylsilyl derivatization. The method exhibited good linearity over the concentration range of 0.5–10.0 ng/mL (r² = 0.992) and achieved a LOD of 0.1 ng/mL. Recovery ranged from 35.3% to 50.8%, while within-day and day-to-day precision values remained below 11.8%. The applicability of the method was demonstrated through successful quantification of TTX in plasma samples obtained from rats following intraperitoneal administration of the toxin, yielding concentrations between 6.55 and 9.49 ng/mL. In another study Man et al. (Man et al. 2010) developed and validated a GC-MS method for the screening and quantification of TTX in pufferfish tissues. The procedure involved extraction with 0.1% acetic acid, alkaline hydrolysis of TTX to its characteristic C9-base, liquid–liquid extraction with n-butanol, and derivatization using N-methyl-N-trimethylsilyltrifluoroacetamide (MSTFA). GC-MS analysis was performed in selective ion monitoring mode using m/z 392 as the quantifier ion and salicylic acid as the internal standard. The assay exhibited excellent linearity (R² ≥ 0.998), an average recovery of 98.4%, and LOD and LOD of 0.05 and 0.1 ng on-column, respectively. The method was successfully applied to several Malaysian pufferfish species, revealing TTX concentrations ranging from 1.34 to 4.11 µg/g muscle tissue in toxic species. To improve the sensitivity of GC-MS-based TTX determination, Stan’kov et al. (Stan’kov et al. 2016) converted TTX into its characteristic C9-base hydrolysis product prior to GC-MS detection performed in SIM mode (m/z 407, 392, and 376). The method achieved a LOD of 0.001 µg/mL in blood plasma. The analytical range was 0.05–1.0 µg/mL, and the assay demonstrated good reproducibility and applicability to biological and pharmaceutical matrices.

The gas chromatography-based methods developed for TTX analysis are summarized in Table 4. Particular emphasis is placed on derivatization strategies, analytical sensitivity, recovery, and applications to biological and seafood samples.

**Table 4:** Gas chromatography-based methods used for TTX determination

Authors	Technique	Matrix	Sample preparation / IS	LOD / LOQ	Comments
Suenaga and Kotoku (1980)	GC	Stomach contents, serum	Alkaline conversion to C9-base, trimethylsilylation	LOD 0.4 µg/sample	Recoveries 83.7% (stomach contents) and 65.3% (serum)
Kurono et al. (2001)	GC-MS	Plasma	SPE, alkaline conversion to C9-base, TMS derivatization	LOD 0.1 ng/mL	Linearity 0.5–10 ng/mL; recoveries 35.3–50.8%
Man et al. (2010)	GC-MS	Pufferfish tissues	MSTFA derivatization; salicylic acid IS	LOD 0.05 ng / LOQ 0.1 ng	Recoveries 98.4%; TTX 1.34–4.11 µg/g
Stan’kov et al. (2016)	GC-MS	Plasma, pharmaceutical matrices	C9-base conversion	LOD 0.001 µg/mL	Analytical range 0.05–1.0 µg/mL

### Biological and immunochemical detection of tetrodotoxin

Biological and immunochemical methods have long played an important role in the detection of TTX, particularly as rapid screening tools for food safety monitoring and clinical investigations. Historically, the mouse bioassay (MBA) served as the reference method for TTX detection and regulatory control of marine toxins (Noguchi and Mahmud 2001). The assay relies on the characteristic neurological symptoms and time-to-death observed in mice following administration of toxin-containing extracts. Despite its simplicity and ability to estimate overall biological toxicity, the MBA suffers from limited specificity, substantial inter-laboratory variability, ethical concerns related to animal use, and an inability to distinguish TTX from structurally related analogues or other neurotoxins (Reverté et al. 2018). These limitations have largely driven the transition toward alternative analytical approaches.

Among immunochemical techniques, enzyme-linked immunosorbent assay (ELISA) represent the most extensively developed platform for TTX screening. Early competitive enzyme immunoassays by Kawatsu et al., (Kawatsu et al. 1997) employing monoclonal antibodies raised against TTX–protein conjugates achieved working ranges between approximately 2 and 100 ng/mL and demonstrated good correlation with mouse bioassays. In another study by Zhong et al. (Zhong et al. 2012), ELISA further improved analytical performance, reporting working ranges of 5–108 ng/mL with acceptable intra- and inter-assay precision. More recently, matrix-adapted ELISA methods have been successfully applied by Reverté et al. (Reverté et al. 2018) to shellfish tissues, including oysters and mussels, where LOD typically ranged from 20 to 50 µg/kg after accounting for extraction recovery and matrix effects. Further advances by Campàs et al. (Campàs et al. 2020), have led to the development of magnetic bead-based immunoassays, which combine competitive antibody recognition with colorimetric detection. Such methods have achieved effective detection limits as low as 1 µg/kg in oysters and razor clams and approximately 3 µg/kg in mussels.

While ELISA-based methods provide sensitive and relatively inexpensive screening, their multi-step protocols and dependence on laboratory infrastructure have stimulated the development of more rapid point-of-care formats. Lateral-flow immunochromatographic assays (LFIAs) have emerged as attractive alternatives for rapid on-site detection. Conventional colloidal gold strips provide qualitative results within minutes, whereas more advanced platforms have substantially improved analytical sensitivity. For example, a quantum dot nanobead/gold nanoflower-based LFIA developed by Shen et al. (Shen et al. 2017) achieved a LOD of 0.2 ng/mL, while an aptamer–antibody dual-recognition LFIA reported by Tang et al. (Tang et al. 2025) reached an LOD of 8 ng/mL and enabled smartphone-assisted semi-quantitative analysis.

Beyond portable lateral-flow devices, optical biosensor platforms have attracted increasing attention owing to their potential for highly sensitive, quantitative, and real-time toxin detection. Surface plasmon resonance (SPR)-based immunosensors and planar waveguide optical biosensors represent another important category of immunochemical detection methods. Taylor et al. (Taylor et al. 2011) developed an inhibition-based SPR immunoassay for the determination of TTX in clinically and toxicologically relevant matrices, including pufferfish liver and muscle extracts as well as human urine. After optimization of assay conditions to minimize matrix effects, the method achieved LOD corresponding to approximately 1 ng/mL in pufferfish liver extracts, 6 ng/mL in muscle extracts, and 17 ng/mL in urine samples. A notable advancement was subsequently reported by Yakes et al. (Yakes et al. 2014), who developed the method which enabled direct detection of TTX in pufferfish matrix with a LOD of 0.09 ng/mL and an analysis time of approximately 3.5 min.

Recent advances in portable biosensor technologies have further expanded the possibilities for rapid on-site TTX screening. Liu et al. (Liu et al. 2024) developed a smartphone-based fluorescent immunosensor utilizing antibody-functionalized cotton swabs and fluorescent quantum dot-loaded metal–organic framework (QD@MOF*Ab) biocomposites. The assay employs a sandwich immunorecognition format in which TTX captured on antibody-immobilized swabs is subsequently detected using fluorescent MOF-based probes. The method achieved a visual detection limit of 0.4 ng/mL, while smartphone-assisted quantification provided a lower LOD of 0.13 ng/mL over a working range of 0.2–400 ng/mL.

Further advances in integrated biosensor design were reported by Zhang et al. (Zhang et al. 2026), who developed a nanozyme-catalyzed colorimetric magnetic microfluidic immunosensor (Nano-CMI) for rapid on-site TTX detection. The platform combines an aptamer–antibody sandwich recognition strategy with nanozyme-mediated signal amplification and magnetic microfluidic handling on a six-chamber chip. TTX is captured by aptamer-functionalized magnetic beads and subsequently recognized by antibody-conjugated AuNR@Pt@m-SiO2 nanozymes, which catalyze a colorimetric reaction detectable by smartphone-based RGB image analysis. The assay enabled quantitative determination of TTX over a concentration range of 0.2–20 ng/mL with a LOD of 0.2 ng/mL. The method demonstrated high selectivity against other marine toxins, including saxitoxin, okadaic acid, and domoic acid, as well as satisfactory recoveries ranging from 91.8% to 105.2% in spiked water samples.

A different biosensing strategy was recently proposed by Reverté et al. (Reverté et al. 2026) who developed an automated patch clamp (APC) single-cell biosensor for the simultaneous detection of TTX and paralytic shellfish toxins (PSTs) in shellfish. Unlike antibody-based assays, the method relies on direct measurement of toxin-induced inhibition of voltage-gated sodium channel currents in Neuro-2a cells. The biosensor was capable of detecting both parent toxins and their toxic analogues, providing an integrated assessment of overall neurotoxicity. Validation using 73 naturally contaminated shellfish samples demonstrated excellent agreement with reference LC-MS/MS and LC-HRMS methods (*R* = 0.9985), including samples containing co-occurring PSTs and TTX analogues.

Despite their high sensitivity and operational simplicity, biological and immunochemical methods remain susceptible to matrix effects, antibody cross-reactivity, variability in extraction recovery, and limited ability to discriminate among individual TTX analogues. Consequently, these techniques are primarily used as screening tools, whereas positive findings generally require confirmation by chromatographic methods coupled with mass spectrometry. Nevertheless, the continuous development of highly specific antibodies, novel recognition elements, optical sensing technologies, and functional cell-based biosensors has significantly improved the analytical capabilities of biological and immunochemical TTX detection and expanded its applicability for both food safety monitoring and clinical toxicology.

### Risk assessment and regulatory framework

Risk assessment of TTX has evolved considerably over the last decade, driven largely by the unexpected detection of TTX and its analogues in shellfish harvested from European waters (Katikou 2019). While the toxin has long been recognized as a major food safety concern in Asian countries due to its association with pufferfish poisoning, the emergence of TTX in bivalve molluscs and gastropods from temperate regions has prompted renewed attention from regulatory authorities and risk assessors worldwide. Despite increasing reports of TTX occurrence in marine organisms, the current risk assessment framework remains focused almost exclusively on acute toxicity, reflecting the rapid onset and potentially life-threatening nature of TTX intoxication. The most influential toxicological evaluation to date was conducted by the European Food Safety Authority (EFSA) in 2017 (EFSA Panel on Contaminants in the Food Chain (CONTAM) et al. 2017). Following a comprehensive review of available toxicological and exposure data, the EFSA CONTAM Panel established a group acute reference dose (ARfD) of 0.25 µg/kg body weight for TTX and its analogues. This value was derived from a no-observed-adverse-effect level (NOAEL) of 25 µg/kg body weight identified in an acute oral mouse study and the application of a default uncertainty factor of 100 to account for interspecies and intraspecies variability. Although human poisoning cases suggested that severe neurological symptoms may occur following ingestion of approximately 0.2 mg TTX, the uncertainties associated with dose reconstruction and exposure estimation precluded their direct use in quantitative risk assessment. Nevertheless, these clinical observations were considered broadly consistent with the ARfD derived from animal data. To translate toxicological thresholds into practical food safety guidance, EFSA combined occurrence data from European shellfish monitoring programmes with a conservative large-portion consumption scenario of 400 g shellfish meat. Based on this assessment, a concentration below 44 µg TTX equivalents/kg shellfish meat was considered unlikely to result in adverse health effects in consumers. Importantly, this value was not proposed as a legally enforceable maximum limit but rather as a health-based guidance level intended to support risk management decisions and monitoring programmes. Consequently, several European countries have adopted the 44 µg/kg threshold as a provisional reference value. The Netherlands, for example, initially implemented a more precautionary action level of 20 µg/kg before subsequently aligning its monitoring strategy with the EFSA-derived threshold (Gerssen et al. 2018). Despite these advances, regulatory approaches toward TTX remain highly heterogeneous. Japan, where TTX poisoning has historically been associated with pufferfish consumption, employs a well-established regulatory framework that includes strict control of fish species, fishing areas, edible tissues, and food preparation practices (Lago et al. 2015). Within the European Union, tetrodotoxin TTX is not currently subject to a specific maximum permitted level in seafood. Nevertheless, Regulation (EC) No. 853/2004 indirectly addresses the risk of TTX poisoning by prohibiting the placement on the market of fishery products derived from poisonous fish belonging to the families *Tetraodontidae*,* Diodontidae*,* Molidae*, and *Canthigasteridae*, which include species known to accumulate TTX (Regulation (EC) 2004).

A major challenge for future risk assessment is the substantial uncertainty surrounding chronic exposure and the toxicological relevance of the growing number of identified TTX analogues (Malykin et al. 2024). Current health-based guidance values rely predominantly on acute animal studies, while information regarding long-term effects, developmental toxicity, susceptible population groups, and potential interactions with co-occurring marine biotoxins remains limited. Furthermore, relative potency factors have been established for only a small number of analogues, complicating the interpretation of occurrence data and the estimation of total toxic equivalents. Climate-driven changes in marine ecosystems, including the expansion of TTX-producing microorganisms, geographical spread of vector species (Meng et al. 2024), or even temperature of water may further influence future exposure patterns and necessitate periodic reassessment of current risk estimates (Dhanji-Rapkova et al. 2023).

Consequently, international expert bodies continue to emphasize the need for harmonized analytical methodologies, certified reference materials, comprehensive occurrence monitoring, and additional toxicological investigations (Varini et al. 2025). The generation of robust data on analogue toxicity, chronic exposure, and environmental drivers of TTX accumulation will be essential for refining existing risk assessments and supporting the development of scientifically justified regulatory standards. Until such data becomes available, the EFSA ARfD and the associated guidance level of 44 µg TTX equivalents/kg shellfish meat remain the principal benchmarks for evaluating consumer safety and managing the public health risks associated with TTX contamination (EFSA Panel on Contaminants in the Food Chain (CONTAM) et al. 2017).

## Conclusions and future directions

Current evidence firmly establishes TTX as one of the most potent naturally occurring neurotoxins and a unique pharmacological probe for voltage-gated sodium channels. Over recent decades, substantial progress has been made in understanding its chemical structure, mechanism of action, ecological distribution, toxicology, and analytical detection. TTX has now been identified in an expanding range of marine and terrestrial organisms, including species inhabiting temperate regions, highlighting its growing relevance for food safety and public health. At the same time, analytical methodologies have evolved from relatively nonspecific bioassays to highly sensitive and selective LC–MS/MS-based approaches, greatly improving the detection and quantification of TTX and selected analogues in food, environmental, and biological specimens. Clinical studies have also suggested an analgesic signal for TTX in cancer-related and chemotherapy-induced neuropathic pain, although its therapeutic efficacy and long-term safety require confirmation in larger, adequately powered clinical trials.

Despite these advances, several important questions remain unresolved. The complete biosynthetic pathway of TTX has not yet been elucidated, and although current evidence supports the involvement of bacterial symbionts and trophic accumulation, the relative contribution of microbial and host-derived processes remains uncertain. Considerable knowledge gaps also persist regarding the toxicological significance of many naturally occurring TTX analogues, including their relative toxic potencies, toxicokinetics, and contribution to overall toxicity. From an analytical perspective, reliable quantification continues to be challenged by the extreme polarity of TTX, low concentrations in biological specimens, limited availability of certified reference standards for many analogues, and the complexity of biological and food matrices. Furthermore, although EFSA has proposed a health-based guidance value for TTX in shellfish, additional occurrence, toxicological, and exposure data are required to refine future risk assessments and support evidence-based regulatory decision-making.

Future research should therefore prioritize clarification of TTX biosynthesis through integrated genomic, microbiological, and biochemical approaches, while expanding ecological surveillance to better understand the influence of environmental change on toxin occurrence and distribution. Continued efforts are also needed to establish toxic equivalency factors for a broader range of TTX analogues, generate robust human toxicokinetic data, and improve understanding of dose–response relationships across different populations. Equally important is the further harmonization and validation of analytical methodologies, including expanded availability of certified reference materials, interlaboratory quality-assurance programs, and standardized confirmatory LC–MS/MS or LC–HRMS workflows. Emerging biosensor technologies represent promising tools for rapid screening and sample prioritization; however, confirmatory analysis using validated chromatographic–mass spectrometric methods remains essential for reliable clinical, food safety, and forensic investigations. From a clinical and forensic perspective, timely specimen collection and appropriate matrix selection are critical because circulating TTX concentrations decline rapidly following exposure. Whenever possible, urine, gastric contents, vomitus, and suspected food remnants should complement blood analysis, particularly when sampling is delayed. Collectively, these priorities will improve the detection, interpretation, and management of TTX exposure while strengthening preparedness for this increasingly important marine toxin from both public health and forensic perspectives.

## Data Availability

No datasets were generated or analysed during the current study.

## Abbreviations

BEH: Bridged ethylene hybrid
CE: Capillary electrophoresis
Cmax: Maximum plasma concentration
CONTAM: EFSA panel on contaminants in the food chain
CZE: Capillary zone electrophoresis
DAD: Diode array detector
dd-MS²: Data–dependent tandem mass spectrometry
EC: European commission
EFSA: European food safety authority
ELISA: Enzyme–linked immunosorbent assay
ESI: Electrospray ionization
FA: Formic acid
FDA: Food and drug administration
FLD: Fluorescence detection
GC-MS: Gas chromatography–mass spectrometry
GC-MS/MS: Gas chromatography–tandem mass spectrometry
HILIC: Hydrophilic interaction liquid chromatography
HPLC: High–performance liquid chromatography
HRMS: High–resolution mass spectrometry
IS: Internal standard
LC: Liquid chromatography
LC–ESI-MS: Liquid chromatography–electrospray ionization mass spectrometry
LC-FLD: Liquid chromatography with fluorescence detection
LC-HRMS: Liquid chromatography–high–resolution mass spectrometry
LC-MS: Liquid chromatography–mass spectrometry
LC-MS/MS: Liquid chromatography–tandem mass spectrometry
LLOQ: Lower limit of quantification
LOD: Limit of detection
LOQ: Limit of quantification
MCX: Mixed–mode cation exchange
MRM: Multiple reaction monitoring
MS: Mass spectrometry
MS/MS: Tandem mass spectrometry
Nav: Voltage–gated sodium channel
NOAEL: No observed adverse effect level
ODS: Octadecylsilane
PBK: Physiologically based kinetic
Q-Exactive Orbitrap HR-MS: Quadrupole orbitrap high–resolution mass spectrometry
QuEChERS: Quick, easy, cheap, effective, rugged and safe
RP-HPLC UV: Reversed–phase high–performance liquid chromatography with ultraviolet detection
RSD: Relative standard deviation
SIM: Selected ion monitoring
SPE: Solid–phase extraction
SPME: Solid–phase microextraction
SPR: Surface plasmon resonance
SRM: Selected reaction monitoring
TTX: Tetrodotoxin
UHPLC: Ultra–high–performance liquid chromatography
UPLC: Ultra–performance liquid chromatography
UV: Ultraviolet
VGSC: Voltage–gated sodium channel
WCX: Weak cation exchange
